# What sharks and mammals share

**DOI:** 10.7554/eLife.80392

**Published:** 2022-07-01

**Authors:** Sophie Pantalacci

**Affiliations:** 1 Laboratoire de Biologie et Modélisation de la Cellule, Ecole Normale Supérieure de Lyon, CNRS, UMR 5239, Inserm, U1293, Université Claude Bernard Lyon 1 Lyon France

**Keywords:** shark, tooth development, morphogenesis, gene expression, dental evolution, Other, Scyliorhinus canicula

## Abstract

The tooth shape of sharks and mice are regulated by a similar signaling center despite their teeth having very different geometries.

**Related research article** Thiery AP, Standing AS, Cooper RL, Fraser GJ. 2022. An epithelial signalling centre in sharks supports homology of tooth morphogenesis in vertebrates. *eLife* **11**:e73173. doi: 10.7554/eLife.73173.

The teeth of a mouse, a fish and a reptile may look wildly different from each other, but they have all evolved from a common ancestor which lived about 500 million years ago (Fraser et al., 2010). In vertebrates with a jaw, a tooth emerges from a bended epithelium that encloses a pool of cells – the mesenchyme – which take the form of the future tooth. This shape is then consolidated when the enamel and the dentin, respectively produced at the interface of the epithelium and the mesenchyme, become mineralized.

While the genes that control when and where teeth are created are the same in most vertebrate lineages (Jernvall and Thesleff, 2012; Tucker and Fraser, 2014), what controls the incredible variety of tooth shapes is less clear. The number and geometry of the ‘tips’ (or cusps) a tooth contains can vary greatly within and across different vertebrates. For instance, incisors contain a single primary cusp projecting from their crown, whereas molars are more complex and contain additional secondary cusps. Simple, one-cusped teeth were first to emerge during evolution, while more complex teeth with multiple cusps arose independently in different lineages; it is therefore possible that distant vertebrates evolved different solutions to form complex tooth shapes.

Much of what is known about how teeth are shaped has come from studying mice. This has revealed that during mammalian development, transient signaling centers known as enamel knots regulate tooth shape by releasing factors that alter the behavior of nearby cells (Jernvall and Thesleff, 2012; Jernvall et al., 1994). Depending on their vicinity to the signaling center, cells will adopt different behaviors; cells within and near the knot stop multiplying and differentiate, while those further away proliferate.

A primary enamel knot forms first and initiates the folding that will shape the tooth’s crown. Then, for more complex teeth like molars, secondary enamel knots develop sequentially and regulate the growth and differentiation of each cusp, a process which takes place from tip to bottom (Figure 1). In mice, the primary enamel knot and the first secondary enamel knot physically overlap, but how this transition takes place remains unclear (Du et al., 2017).

**Figure 1.**
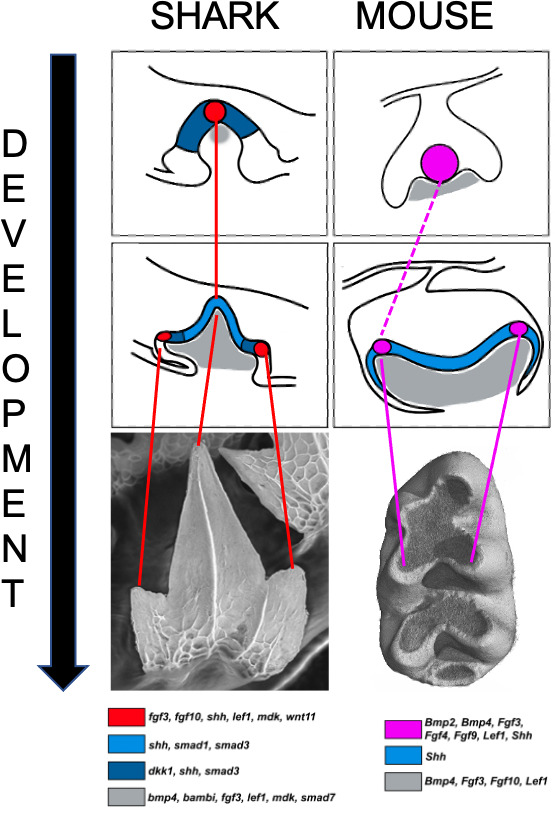
Signaling centers and tooth shape in catsharks and mice. Teeth in catsharks (left) and lower molars in mice (right) both have signaling centers (red and pink circles respectively) that organize the shape of teeth during development. These centers reside at the tip of emerging cusps; they appear sequentially as the tooth develops, releasing signaling factors which activate genes in the neighboring epithelium (blue) and the underlying mesenchyme (gray) that will take the form of the future tooth. The mouse primary enamel knot may give rise to the secondary enamel knot (dashed line) associated with the primary cusp. The schematics are based on histological sections which are shown below, along with a color code representing the expression pattern of several genes activated during tooth development.

Image credit: Image adapted from Figure 8 and 10 from Thiery et al.; mouse molar picture from Sophie Pantalacci (CC BY 4.0).

While the enamel knot was first detected in the early 20th century, it has only been identified as a signaling center that shapes mammalian teeth in the last 30 years. When the developing teeth of reptiles, bony fish and sharks were examined, many of the genes responsible for the signals released from the enamel knot were also shown to be activated at certain sites. But differences in the pattern of gene expression, combined with missing histological criteria, suggested that these centers may not be homologous to those found in mammals (Debiais-Thibaud et al., 2015; Richman and Handrigan, 2011). Now, in eLife, Alexandre Thiery, Ariane Standing, Rory Cooper and Gareth Fraser report a signaling center in the developing molar teeth of catsharks which clearly resembles the enamel knot (Thiery et al., 2022).

First, the team (who are based at the University of Florida, University of Sheffield, University of Geneva and King’s College London) examined which genes are expressed in the developing teeth of shark embryos. This revealed that some of the genes switched on in the mammalian enamel knots are also activated in the epithelium that coats the tip of emerging primary and secondary cusps in sharks (Figure 1). In addition, the genes activated by the signals emitted from the enamel knot were expressed in the mesenchymal tissue underlying the epithelium. Strikingly, differentiation was found to progress downwards, with cells at the tip ceasing proliferation and maturing before the cells beneath them. This tip-to-bottom differentiation, along with the presence of a signaling center in each cusp, resembles what happens in mammalian molars.

Next, in a technically challenging feat, Thiery et al. used pharmacological treatments to perturb the Wnt signaling pathway, which plays a key role in forming the enamel knots in mice. This reduced the size of the sharks’ teeth and the number of cusps, suggesting that the Wnt pathway may also regulate tooth shape in sharks.

Finally, Thiery et al. studied an in silico model of tooth morphogenesis which can recapitulate the varied molar structures found in seals. In the simulation, a diffusing activator triggers the formation of secondary enamel knots, which then emit local inhibiting signals that prevent other knots from forming in the vicinity. Secondary knots also release a differentiation factor to locally stop proliferation and activate cusp morphogenesis and differentiation. Together with mechanical forces from the mesenchyme, these epithelial regulations determine the sequence of secondary enamel knot formation as well as cusp morphogenesis and differentiation, from the primary cusp tip down towards the bottom of the tooth (Salazar-Ciudad and Jernvall, 2002; Salazar-Ciudad, 2008). With just a small number of modifications, this model could also form catshark-looking teeth, which is not surprising given that catshark teeth and seal molars have a similar shape.

Manipulating the activation-inhibition parameters in the model allowed Thiery et al. to increase or decrease the number of cusps, as well as to recreate the varied tooth shapes seen at different positions during catsharks’ lifetime. The model could recapitulate some but not all aspects of pharmacologically perturbed teeth. Pinpointing the parameters that need to be modified for the model to generate these additional phenotypes could provide further insights into the differences between tooth development in sharks and mammals.

In conclusion, Thiery et al. propose that a signaling center similar to the enamel knot regulates the development of shark teeth. Intriguingly, this structure may even predate the advent of teeth as it resembles the signaling center in the tough scales coating the exterior of sharks, which supposedly appeared before teeth during evolution (Fraser et al., 2010). The findings of Thiery et al. help to pave the way for future studies that can characterize these signaling centers in several vertebrate groups and establish potential lineage-specific features for these signaling centers and the spatio-temporal context in which they operate. This will establish the degree to which complex teeth that arose through a different evolutionary trajectory use similar mechanisms.

Characterizing the signaling center of other vertebrate lineages may also help to answer questions about the way enamel knots are arranged over time in the highly specialized teeth of mice, which has been the subject of heated debate (Ahtiainen et al., 2016; Du et al., 2017; Hovorakova et al., 2016; Mogollón et al., 2021; Sadier et al., 2019). Although research conducted on mice has so far served as a reference for tooth development in other vertebrates, there is perhaps much to learn if the students become the teachers.

## References

[bib1] Ahtiainen L, Uski I, Thesleff I, Mikkola ML (2016). Early epithelial signaling center governs tooth budding morphogenesis. The Journal of Cell Biology.

[bib2] Debiais-Thibaud M, Chiori R, Enault S, Oulion S, Germon I, Martinand-Mari C, Casane D, Borday-Birraux V (2015). Tooth and scale morphogenesis in shark: an alternative process to the mammalian enamel knot system. BMC Evolutionary Biology.

[bib3] Du W, Hu JKH, Du W, Klein OD (2017). Lineage tracing of epithelial cells in developing teeth reveals two strategies for building signaling centers. The Journal of Biological Chemistry.

[bib4] Fraser GJ, Cerny R, Soukup V, Bronner-Fraser M, Streelman JT (2010). The odontode explosion: the origin of tooth-like structures in vertebrates. BioEssays.

[bib5] Hovorakova M, Lochovska K, Zahradnicek O, Domonkosova Tibenska K, Dornhoferova M, Horakova-Smrckova L, Bodorikova S (2016). One odontogenic cell population contributes to the development of the mouse incisors and of the oral vestibule. PLOS ONE.

[bib6] Jernvall J, Kettunen P, Karavanova I, Martin LB, Thesleff I (1994). Evidence for the role of the enamel knot as a control center in mammalian tooth cusp formation: non-dividing cells express growth stimulating Fgf-4 gene. The International Journal of Developmental Biology.

[bib7] Jernvall J, Thesleff I (2012). Tooth shape formation and tooth renewal: evolving with the same signals. Development.

[bib8] Mogollón I, Moustakas-Verho JE, Niittykoski M, Ahtiainen L (2021). The initiation knot is a signaling center required for molar tooth development. Development.

[bib9] Richman JM, Handrigan GR (2011). Reptilian tooth development. Genesis.

[bib10] Sadier A, Twarogowska M, Steklikova K, Hayden L, Lambert A, Schneider P, Laudet V, Hovorakova M, Calvez V, Pantalacci S (2019). Modeling *Edar* expression reveals the hidden dynamics of tooth signaling center patterning. PLOS Biology.

[bib11] Salazar-Ciudad I, Jernvall J (2002). A gene network model accounting for development and evolution of mammalian teeth. PNAS.

[bib12] Salazar-Ciudad I, Schatten G (2008). Current Topics in Developmental Biology.

[bib13] Thiery AP, Standing AS, Cooper RL, Fraser GJ (2022). An epithelial signalling centre in sharks supports homology of tooth morphogenesis in vertebrates. eLife.

[bib14] Tucker AS, Fraser GJ (2014). Evolution and developmental diversity of tooth regeneration. Seminars in Cell & Developmental Biology.

